# Case report: Long-term survival in a patient with metastatic colorectal cancer treated with trifluridine/tipiracil in the third-line setting

**DOI:** 10.3389/fonc.2023.1112224

**Published:** 2023-03-01

**Authors:** Mohamed ELBassiouny

**Affiliations:** Department of Clinical Oncology and Nuclear Medicine, Ain Shams University, Cairo, Egypt

**Keywords:** long-term survival, metastatic colorectal cancer, trifluridine/tipiracil, case report, third-line

## Abstract

Third-line treatment with trifluridine/tipiracil (FTD/TPI) is recommended for patients with metastatic colorectal cancer (mCRC) or gastric/gastroesophageal cancer (GC) who have progressed beyond first- and second-line therapy. We describe a patient with long-term survival following treatment with FTD/TPI. The patient, a 70-year-old woman diagnosed with right-sided mCRC (T3/N1) with metastases to the aortocaval and precaval lymph nodes, received first-line panitumumab and capecitabine for 6 months, followed by second-line bevacizumab and oxaliplatin. She had disease progression following 9 months of second-line therapy and began third-line treatment with FTD/TPI (50 mg bid). Three months after treatment initiation, lymph node involvement was reduced, and following 12 months of FTD/TPI treatment, her disease had stabilized, and she reported no treatment-related adverse events. She remained on the same dose of FTD/TPI for more than 27 months after initiating treatment, with maintenance of stable disease. This patient with mCRC demonstrated a survival benefit with FTD/TPI beyond those reported in published clinical trial data and real-world studies.

## Introduction

Colorectal cancer (CRC) is among the most frequently diagnosed malignancies in the world and presents a substantial health burden (1). For patients with metastatic CRC (mCRC) who have progressed beyond first- and second-line therapy, treatment options are limited (2, 3). According to the ESMO guidelines, third-line options for mCRC include the chemotherapeutic agent trifluridine/tipiracil (FTD/TPI) (4). The pivotal RECOURSE trial in mCRC showed significant median overall survival (mOS) improvement from 5.3 months with placebo to 7.1 months with FTD/TPI (5). In extensive real-world studies, FTD/TPI provided a median progression-free survival of 2.8 months when used as a later-line therapy for mCRC (6). Published cases have shown long-term survival and maintenance of quality of life (QoL) with FTD/TPI as late-line chemotherapy in other recurrent gastrointestinal cancers (7, 8). Here, we report the details of an Egyptian patient with mCRC with long-term survival following treatment with FTD/TPI.

## Case description

A 70-year-old woman (height 160 cm; weight 55 kg) with hypertension was diagnosed with right-sided mCRC (T3/N1) on February 2019, with metastases to the aortocaval and precaval lymph nodes (18 mm x 13 mm, standardized uptake volume [SUV] 16). She had KRAS-wild-type mCRC (and no other detected mutations associated with CRC) with carcinoembryonic antigen (CEA) levels of 35 ng/ml. The patient underwent a hemicolectomy and commenced treatment with panitumumab (400 mg by intravenous infusion every two weeks) and capecitabine (2 x 500 mg tablets every 12 hours) but dose was reduced by 25% due to her fragility. Assessed with positron emission tomography (PET) scans and CEA evaluations, the patient had a regressive course after 3 months of treatment according to a stabilized lymph node size, reduced SUV (5.5) and reduction of CEA levels to 9.7 ng/ml. However, 6 months after starting therapy, the lymph node size had increased (33 mm x 33 mm, SUV 11) and CEA levels had increased to 176 ng/ml indicating disease progression.

The patient commenced second-line therapy with bevacizumab (400 mg by intravenous infusion every two weeks) and oxaliplatin (100 ml by intravenous infusion every two weeks) in September 2019. Disease regressed 3 months later: aortocaval lymph node was no longer visible, the precaval lymph node had decreased in size (12 mm x 25 mm, SUV 9), and CEA levels had decreased to 39 ng/ml. Disease remained stable after a further 3 months of treatment with bevacizumab and oxaliplatin (20 mm x 22 mm, SUV 3.6, CEA levels of 11 ng/ml), but 3 months after this (following a change in therapy to bevacizumab plus capecitabine, due to neurotoxicity and fatigue that was attributed to oxaliplatin; and a total of 9 months on second-line therapy) the existing lymph node involvement increased in size (25 mm x 25 mm, SUV 10.4), new lymph node involvement was detected and CEA levels increased slightly to 24 ng/ml. As a result, treatment with bevacizumab and irinotecan was initiated (June 2020), but was discontinued after one cycle due to alopecia, neutropenia and general weakness.

In July 2020, she began third-line treatment with FTD/TPI (50 mg bid), to control disease progression. After 3 months of treatment, lymph nodes had decreased in size (14 mm, SUV 4) and CEA levels had almost normalized at 2.8 ng/ml. Following a further 6 months of treatment with FTD/TPI (with no dose delays or reductions), all lymph nodes had disappeared, with the exception of the precaval lymph node (20 mm, SUV 17), but CEA levels had increased. By July 2021 (12 months receiving FTD/TPI as third-line treatment) her disease had stabilized and CEA levels were 58 ng/ml. The patient experienced no adverse reactions related to FTD/TPI treatment. The patient remains on the same dose of FTD/TPI >27 months after initiating this treatment and continues to have stable disease. As of March 2022, CEA level was 11.7 ng/ml, and the patient remains on FTD/TPI treatment as of October 2022. All treatments and outcomes are summarized in **Table 1**.

**Table 1:** Treatment timeline and responses.

Date	Treatment line	Treatment	Response	Lymph nodes, mm	SUV	CEA, ng/ml
**Feb 2019**	1L	Panitumumab (400 IV q2wk) + capecitabine (2 x 500 mg oral q12hrs); dose reduced by 25% due to fragility	NR	18 x 13	16	35
**May 2019**			Regression	Size stabilized	5.5	9.7
**Aug 2019**			Progression	33 x 33	11	176
**Sept 2019**	2L	Bevacizumab (400 mg IV q2wk) and oxaliplatin (100 ml IV q2wk)	NR	NR	NR	NR
**Dec 2019**			Regression	Aortocaval node no longer visible; precaval lymph node decreased in size (12 x 25)	9	39
**Mar 2020**			Stable disease	20 x 22	3.6	11
**Jun 2020**		Bevacizumab plus capecitabine	Progression	Existing node increased in size (25 x 25) and a new node was detected	10.4	24
**Jun 2020**		Bevacizumab + irinotecan initiated; discontinued after one cycle due to alopecia, neutropenia and weakness	NR	NR	NR	NR
**Jul 2020**	3L	FTD/TPI (50 mg oral bid)	NR	NR	NR	NR
**Oct 2020**			Regression	Nodes had decreased in size (14)	4	Normalized at 2.8
**Apr 2021**			Regression	All nodes had disappeared, except precaval node (20)	17	CEA levels increased
**Jul 2021**			Stable disease	NA	NA	58
**Mar 2022**				NA	NA	11.7
**Oct 2022**				NA	NA	NA

CEA, carcinoembryonic antigen; IV, intravenous; NA, not available; NR, not relevant; q12hrs, every 12 hours; q2wk, every 2 weeks; SUV, standardized uptake volume.

## Discussion

In this case study, an Egyptian patient with mCRC had an impressive survival benefit with FTD/TPI, in excess of those reported in published clinical trial data and real-world studies. She received first- and second-line treatment for 17 months after diagnosis, but then achieved a partial pathological response with FTD/TPI and was still alive, responding and receiving FTD/TPI more than 27 months later. FTD/TPI has demonstrated long-term efficacy in other case reports of mCRC (9), and other cancers, such as metastatic rectal cancer (8). However, it is unusual for a patient to maintain a response for longer during third-line treatment than during first- or second-line treatment. As an example, in the phase III RECOURSE trial, at 16 months, only 2 of 534 patients in the FTD/TPI arm were progression-free (5). Because this patient had KRAS wild-type disease, this may have contributed to the duration of her survival, as it is known that patients with KRAS wild-type mCRC have a better prognosis (in one study of chemotherapy recipients, mOS was 16.9 months and 6.9 months in patients with KRAS wild-type and KRAS mutation, respectively) (10). Furthermore, the presence of <3 metastatic sites and a time since diagnosis of metastatic disease ≥18 months were positive prognostic factors in the RECOURSE trial, and these are factors that were present in this case. However, the improvements in median progression-free survival (PFS) in the presence of these factors was modest in the RECOURSE trial (5, 11). Additional factors that may influence PFS or OS in patients treated with FTD/TPI include CEA <200 ng/ml, neutrophil-to-lymphocyte ratio <5, the development of grade 3 neutropenia, and the number of previous lines of therapy (12–14).

The outcome with FTD/TPI treatment may potentially be influenced by genetic alterations in, for example, the TYMP gene, which encodes thymidine phosphorylase, the enzyme that is primarily responsible for FTD metabolism and that is inhibited by TPI (15). Mutation of this gene may affect the metabolism of FTD/TPI, increasing the plasma concentration of the drug, and potentially contributing to longer survival. Although this information was not available for this patient, it is feasible that such a mutation may have contributed to the prolonged survival with FTD/TPI treatment.

This patient did not experience any adverse events and did not require any dose delays or reductions with FTD/TPI. The reasons for the absence of any of the adverse events that are frequently observed with FTD/TPI treatment, such as neutropenia and leukopenia (5), are not clear. Long-term survival and maintenance of good QoL with FTD/TPI treatment in heavily pre-treated patients with mCRC has been reported in other case studies (16), and ongoing trials of FTD/TPI in a broad spectrum of tumors may provide further insight into optimizing treatment with FTD/TPI.

Choosing the right treatment from the start [i.e., an anti-EGFR agent and chemotherapy, which may be particularly suited in this older patient with KRAS wild-type CRC (17)] was one of the main reasons for the unique result in this case of ongoing third-line treatment after almost 28 months in a patient with mCRC. Future research to identify common factors that may predict such long-term survival during treatment with FTD/TPI could include prospective trials that are powered to assess survival in specific patient subgroups (such as those with <3 or ≥3 sites of metastases; according to ECOG-PS at baseline; length of time from diagnosis to first metastasis; presence/absence of liver metastasis; tumor sidedness; and baseline molecular profile) with different third-line treatments.

## Data availability statement

The original contributions presented in the study are included in the article/supplementary material. Further inquiries can be directed to the corresponding author.

## Ethics statement

Ethical review and approval was not required for the study on human participants in accordance with the local legislation and institutional requirements. The patients/participants provided their written informed consent to participate in this study. Written informed consent was obtained from the individual(s) for the publication of any potentially identifiable images or data included in this article.

## Author contributions

ME assumes final responsibility for the work, had access to all data, contributed extensively to the writing of this case report, and gave full approval to submit this work for publication.
